# Alpha connectivity and inhibitory control in adults with autism spectrum disorder

**DOI:** 10.1186/s13229-020-00400-y

**Published:** 2020-12-07

**Authors:** Veronica Yuk, Benjamin T. Dunkley, Evdokia Anagnostou, Margot J. Taylor

**Affiliations:** 1 Department of Diagnostic Imaging, The Hospital for Sick Children, 555 University Avenue, Toronto, ON M5G 1X8 Canada; 2 Neurosciences and Mental Health Program, SickKids Research Institute, The Hospital for Sick Children, Toronto, ON Canada; 3 Department of Psychology, University of Toronto, Toronto, ON Canada; 4 Department of Medical Imaging, University of Toronto, Toronto, ON Canada; 5 Department of Neurology, The Hospital for Sick Children, Toronto, ON Canada; 6 Bloorview Research Institute, Holland Bloorview Kids Rehabilitation Hospital, Toronto, ON Canada

**Keywords:** Alpha, Autism, Connectivity, Go/No-go, Inhibition, MEG

## Abstract

**Background:**

Individuals with autism spectrum disorder (ASD) often report difficulties with inhibition in everyday life. During inhibition tasks, adults with ASD show reduced activation of and connectivity between brain areas implicated in inhibition, suggesting impairments in inhibitory control at the neural level. Our study further investigated these differences by using magnetoencephalography (MEG) to examine the frequency band(s) in which functional connectivity underlying response inhibition occurs, as brain functions are frequency specific, and whether connectivity in certain frequency bands differs between adults with and without ASD.

**Methods:**

We analysed MEG data from 40 adults with ASD (27 males; 26.94 ± 6.08 years old) and 39 control adults (27 males; 27.29 ± 5.94 years old) who performed a Go/No-go task. The task involved two blocks with different proportions of No-go trials: Inhibition (25% No-go) and Vigilance (75% No-go). We compared whole-brain connectivity in the two groups during correct No-go trials in the Inhibition vs. Vigilance blocks between 0 and 400 ms.

**Results:**

Despite comparable performance on the Go/No-go task, adults with ASD showed reduced connectivity compared to controls in the alpha band (8–14 Hz) in a network with a main hub in the right inferior frontal gyrus. Decreased connectivity in this network predicted more self-reported difficulties on a measure of inhibition in everyday life.

**Limitations:**

Measures of everyday inhibitory control were not available for all participants, so this relationship between reduced network connectivity and inhibitory control abilities may not be necessarily representative of all adults with ASD or the larger ASD population. Further research with independent samples of adults with ASD, including those with a wider range of cognitive abilities, would be valuable.

**Conclusions:**

Our findings demonstrate reduced functional brain connectivity during response inhibition in adults with ASD. As alpha-band synchrony has been linked to top-down control mechanisms, we propose that the lack of alpha synchrony observed in our ASD group may reflect difficulties in suppressing task-irrelevant information, interfering with inhibition in real-life situations.

## Background

Inhibition is one of the core executive functions that allows an individual to control their attention, thoughts, and behaviour by suppressing processes that hinder or are irrelevant to one’s goals [1]. The extent to which individuals with autism spectrum disorder (ASD) experience difficulties with inhibition has been investigated across a variety of tasks. While there are reports showing some preserved inhibition in this population [2–4], others appear to suggest deficits in inhibitory control in people with ASD [5–8], such as in response inhibition specifically [9–12]. Moreover, as inhibition may underlie working memory [13] and cognitive flexibility [1], impairments in inhibition can have significant downstream effects on more complex behaviours required in everyday life in individuals with ASD, such as in reciprocal conversation [14].

These behavioural reports of inhibition deficits suggest differences in the functioning of the inhibitory control brain network in ASD. This network consists of several right-lateralized frontoparietal regions [15–17], including the inferior frontal gyrus (IFG), insula, supplementary motor area (SMA), anterior cingulate cortex (ACC), and inferior and superior parietal lobules (IPL and SPL), of which the right IFG plays a prominent role [18–21]. Research using magnetoencephalography (MEG), which is sensitive to the timing of neural activity [22], has illustrated that these regions appear to be maximally active between 200 and 400 ms, after stimulus onset [23–25]. Electroencephalographic (EEG) studies have also revealed that peaks in event-related potentials at around 200 and 300 ms are associated with conflict monitoring and motor inhibition, respectively [26–29]. This neural activity consists of oscillations at different frequencies, specifically in the theta (4–7 Hz), alpha (8–14 Hz), beta (15–30 Hz), and gamma (> 30 Hz) bands, each of which plays a certain role in successful inhibition. For instance, greater oscillatory activity or power in the theta band has been observed selectively in trials involving response inhibition [30–32], potentially indicating the monitoring of conflicting responses [33, 34]. Increases in alpha power within brain regions are thought to reflect inhibition of a learned response or of task-irrelevant areas [35–37], while beta oscillations are believed to signify inhibition of a motor response [38–40] and maintenance of an ongoing sensorimotor or cognitive state [41]. Modulation of gamma activity may reflect changes in the balance of local excitatory and inhibitory neural activity that underlies a variety of cognitive functions, including response inhibition and other executive functions [42–47]. However, how these brain regions convey information through long-range synchrony to exert inhibitory control has not been well investigated. Generally, interregional communication and integration of information, as well as top-down control, are thought to be mediated by theta, alpha, and beta oscillations [48–53], whereas gamma oscillations are thought to reflect more local dynamics, as the gamma signal tends to diminish over longer distances [54, 55]. In addition, a few EEG studies have found that theta- and alpha-band synchrony are involved in response inhibition [32, 56]. It is important to better understand how connectivity facilitates inhibition, especially in the context of ASD, as people with ASD have demonstrated reduced long-range connectivity patterns in a wide range of domains, especially in the lower frequency bands [57–60] (see [61] for a review). For instance, in tasks of other executive functions, such as working memory and cognitive flexibility, individuals with ASD exhibit reduced theta- and alpha-band connectivity [62, 63].

Several studies have demonstrated atypical activation and functional connectivity of the inhibition brain network in the ASD population, even when behavioural differences were not observed. Compared to controls, fMRI and MEG studies have found that many individuals with ASD exhibit reduced activation of regions in this network, such as in the right IFG and insula [64], ACC [65–67], and right IPL [68, 69], as well as decreased connectivity between nodes of the inhibition network [65, 66], which may be specific to the alpha band [70], and which may worsen with age [71]. Conversely, a few fMRI investigations have shown increased activity in areas within and outside of the inhibition network [72, 73] and increased connectivity among regions of this network that differed from connectivity patterns in controls [74], suggesting the development of alternative neural mechanisms of inhibitory control in these samples. Furthermore, some studies have found that these differences in activity and connectivity relate to task performance and ASD symptomatology [64, 65, 67, 74]. Taken together, it appears that individuals with ASD have alterations in their recruitment of brain regions responsible for inhibitory control, which correlate with behaviour.

The present study examines functional brain connectivity involved in inhibition in adults with ASD to better understand the relationship between these differences in brain function and the inhibitory difficulties experienced by this population. As considerable evidence has suggested that ASD may be characterized by patterns of altered functional connectivity [61, 75–79], we focused our analyses of inhibitory control in ASD on this aspect of neural function. Only one study with a small *N* (11/group) has explored the specificity of connectivity differences in certain frequency bands [70], even though oscillations at different frequencies are thought to underlie distinct inhibitory processes. This previous work assessed connectivity between specified brain regions implicated in inhibition, but not how they might differentially communicate with the rest of the brain. Thus, we investigated whether adults with ASD, compared to controls, would show differences in whole-brain functional connectivity during a Go/No-go response inhibition task using MEG, which is capable of accurately resolving the timing and frequency of neural activity [22]. Specifically, we examined synchrony among areas throughout the brain that form networks in the theta, alpha, and beta frequency bands between 0–400 ms, post stimulus onset, as this window would capture the onset and peak activation of the inhibitory control network and relevant oscillation frequencies underlying long-range interregional communication. We also explored the relationship between any differences in functional connectivity with task performance and difficulties with inhibition in everyday life to ascertain whether such connectivity differences might contribute to inhibitory control abilities.

## Methods and materials

### Participants

We recruited 45 control adults and 54 adults with ASD between the ages of 18–40 years for this study. We screened for full-scale, two subtest IQ ≥ 70 as measured by the Wechsler Abbreviated Scale of Intelligence (WASI or WASI-II) [80, 81], no premature birth, no MRI or MEG contraindications, and in the control group specifically, no history of developmental, neurological, psychiatric, or psychological disorders. All participants with ASD received a primary diagnosis of ASD from a clinical expert. After standard preprocessing of the MEG data, we excluded five adults with ASD due to poor task performance (≤ 50% on Go trials, or ≤ 50% on No-go trials in the Vigilance condition; see the ‘Go/No-go MEG task’ section for a description of the Vigilance condition), one adult with ASD for missing head localization data for more than half of the recording session, and eight adults with ASD for excessive artefacts in the MEG data, such that < 40 trials (half the total possible number of trials) remained after data preprocessing. Subsequently, six control adults were excluded when matching the groups on age and sex.

Thus, in the final sample, there were 39 control adults and 40 adults with ASD. The two groups did not differ on age (*t*(77) = 0.26, *p* = 0.79), sex (X^2^(1) = 0, *p* = 1), or IQ (*t*(65.28) = 1.01, *p* = 0.32). In the ASD group, we had scores for 37 adults on the Autism Diagnostic Observation Schedule, Generic or Version 2 (ADOS-G or ADOS-2) [82, 83]. Demographic data are presented in Table 1.

**Table 1:** Demographic data

	Control (*N* = 39)	ASD (*N* = 40)
	Mean (SD) or count	Mean (SD) or count
Age	27.29 (5.94)	26.94 (6.08)
Sex	27 M, 12 F	27 M, 13 F
Handedness	33 R, 6 L	35 R, 5 L
Full-scale IQ	114.24 (11.33) Range: [92–157], *n *= 38	110.95 (16.63) Range: [72–136], *n * = 38
ADOS CSS	–	6.95 (2.15) Range: [2–10], *n *= 37

*ADOS* Autism Diagnostic Observation Schedule, *CSS* calibrated severity score

### Experimental design

#### Questionnaires

We asked participants to rate themselves on their executive functioning abilities using the Behavior Rating Inventory of Executive Function, Adult Version (BRIEF-A) [84]. The BRIEF-A is a questionnaire consisting of 75 items that assess abilities in a range of executive functions in everyday life, such as inhibition, working memory, and shifting. Participants were also rated on this questionnaire by an informant, or someone who knew the participant well (i.e., parent, partner, close friend, etc.). *T* scores on the Inhibit scale of the BRIEF-A were taken as measures of their everyday inhibitory control, with higher scores indicating poorer inhibition.

Participants were also asked to complete the Social Responsiveness Scale, Second Version (SRS-2) [85]. The SRS-2 includes 65 items that measure impairments in several social domains associated with ASD, such as cognition, communication, and motivation, as well as restricted interests and repetitive behaviour. Scores on the social and restricted interests/repetitive behaviour subscales can be combined to measure overall severity of ASD symptoms. SRS-2 Total *t* scores were used as a measure of ASD symptom severity in our sample, wherein higher scores represent more severe ASD symptomatology.

#### Go/No-go MEG task

**Fig. 1:**
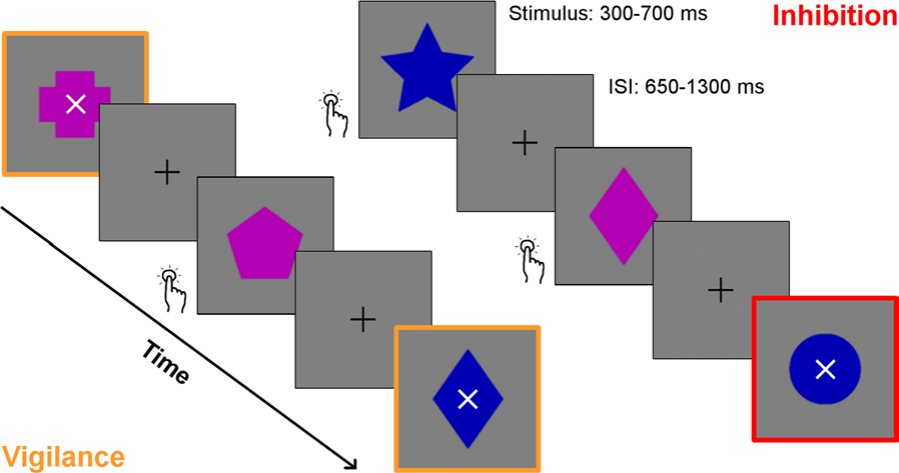
The Go/No-go task. Participants were instructed to press a button as quickly as possible upon seeing the Go stimuli (solid blue or purple shapes) and inhibit this response for No-go stimuli (solid blue or purple shapes with a white ‘x’ in the middle). No-go trials are highlighted in this figure with a coloured border. Stimuli were presented for a duration ranging from 300–700 ms, and interstimulus intervals (ISIs) lasted from 650–1300 ms, with a jitter of ± 200 ms. Stimulus presentation and ISI length were adapted to participants’ performance. Participants completed two blocks of this task: Inhibition (75% Go and 25% No-go trials) and Vigilance (25% Go and 75% No-go trials)

Participants performed a Go/No-go task in the MEG scanner (Fig. 1), adapted from previous work in our lab [24, 25, 69]. They were asked to press a button as quickly as possible in response to Go stimuli, which were five geometric shapes that were either blue or purple, totalling ten possible stimuli. Participants were also instructed to refrain from responding to No-go stimuli, which consisted of the same Go stimuli, but with a white ‘x’ superimposed on the centre of the shape. On each trial, the stimuli appeared in the middle of a grey box measuring 5 × 5 cm, centred on a black background. Between trials, a black fixation cross appeared in the centre of the grey box.

To maintain a rapid response rate, we adapted the stimulus and interstimulus interval (ISI) durations to participants’ performance, as done previously (e.g., [69]). Stimulus duration ranged between 300–700 ms, while ISI duration ranged between 650–1300 ms, plus a random jitter of ± 200 ms; at the beginning of the task, stimulus and ISI durations were at maximum. Durations were increased or decreased within these ranges to maintain an overall accuracy of about 80% in No-go trials and 95% in Go trials. A more detailed description of the protocol for adjusting these durations can be found in Additional file 1.

The task was run in two counterbalanced blocks: Inhibition and Vigilance. In the Inhibition condition, 75% of trials were Go and 25% were No-go, to ensure the establishment of a prepotent response that would have to be inhibited during the No-go trials. In the Vigilance condition, 25% of trials were Go and 75% were No-go, so very little inhibitory control was required for No-go trials. The Vigilance condition was run as a control for the Inhibition condition; while much of the existing literature has compared No-go trials to Go trials, the Go trials contain a strong motor response not present in No-go trials, so we instead contrasted No-go trials from a highly demanding situation (i.e., Inhibition) to those in a less demanding situation (i.e., Vigilance).

Participants were familiarized with both blocks of the task before the MEG session. In the MEG scanner, the stimuli were back-projected onto a screen that was 80 cm away from the dewar and presented using *Presentation 18.1* (Neurobehavioral Systems Inc., https://www.neurobs.com/presentation). Each of the blocks ended when participants successfully completed 80 correct No-go trials or until 10 min had passed.

#### MEG data acquisition

Participants’ MEG data were acquired while lying supine in a 151-channel CTF MEG system (Coquitlam, British Columbia, Canada) inside a magnetically shielded room. Head position was monitored in real time using fiducial coils located on the nasion and the left and right pre-auricular points. Data were sampled at 600 Hz, and a third-order spatial gradient and an anti-aliasing low-pass filter of 150 Hz were applied.

#### MRI data acquisition

Participants’ MRI data were acquired in a 3.0 T MRI scanner (MAGNETOM, Siemens AG, Erlangen, Germany) using a 12-channel head coil. Radio-opaque markers were placed at the MEG fiducial points, allowing for later MEG-MRI co-registration. T1-weighted MRI scans were obtained with the 3D SAG MPRAGE sequence (GRAPPA = 2, TR/TE/FA = 2300 ms/2.96 ms/9º, FOV = 192 × 240 × 256 mm, voxel size = 1.0 mm isotropic).

#### MEG preprocessing

We analysed the MEG data using FieldTrip [86] in MATLAB 2017b (The MathWorks, www.mathworks.com/products/matlab/). Trials were epoched from −1500 to 2000 ms, relative to the onset of the No-go stimulus. Data were filtered offline between 1 and 150 Hz using a fourth-order Butterworth bandpass filter. A notch filter was also applied to remove line noise and its harmonic (60 and 120 Hz). Physiological artefacts were removed by using independent component analysis to decompose the data into independent components, then manually identifying and rejecting components characteristic of eyeblinks and heartbeats. Trials containing signals exceeding 2000 fT or head motion exceeding 5 mm from the median head position were deemed artefactual and removed. Only correct trials were analysed.

To generate the forward model, each participant’s MRI data were co-registered to their MEG data using the fiducials, then used to calculate a subject-specific head model based on the single-shell method [87]. Source activity was estimated at the centre of mass of the 90 AAL atlas regions [88] using a linearly constrained minimum variance beamformer [89] with 5% regularization and centre-of-head bias correction via the neural activity index. A common spatial filter was created using the covariance matrix computed over all trials, through which the entire continuous dataset (after artefact removal) was then projected. This dataset was then epoched as described above.

To assess connectivity, we took the weighted phase lag index (wPLI) between each pairwise connection (excluding those to and from Heschl’s gyrus and olfactory cortex, as the primary auditory and olfactory areas are irrelevant to this task). wPLI is a reliable measure of phase synchronization which is robust to noise and volume conduction artefacts and has demonstrated good statistical power in detecting non-zero phase coherence [90]. wPLI values were calculated over trials using the cross-spectral density matrix, which was computed for signals from − 500 to 1000 ms within each frequency band of interest (theta: 4–7 Hz; alpha: 8–14 Hz; beta: 15–30 Hz) using wavelets with a width of seven cycles. Pairwise wPLI values were transformed into *z* scores using the values in the baseline window (− 500 to 0 ms), then averaged over our time window of interest, 0–400 ms. Connectivity matrices containing these normalized wPLI values were then subjected to statistical analysis to examine within- and between-group differences in network connectivity.

### Statistical analysis

#### Behavioural data

We examined the effects of group (control vs. ASD), rater (self vs. informant) and IQ, as well as the interaction between group and rater, on Inhibit scale scores of the BRIEF-A. We also investigated the effects of group (control vs. ASD), condition (Inhibition vs. Vigilance), age, and IQ, in addition to the interaction between group and condition, on participants’ accuracy on our Go/No-go task. D-prime (d′) was used as a measure of accuracy or ability to distinguish between Go and No-go stimuli when withholding a response. It was calculated by taking the difference between the *z*-transformed hit rate and the *z*-transformed false alarm rate: d′ = *z*(hit rate) *− z*(false alarm rate). Correct No-go trials were considered as hits, while incorrect Go trials were deemed as false alarms. All behavioural data were analysed using linear mixed effects models, as implemented by the nlme package, in R 3.5.0 (R Core Team, https://www.r-project.org/). Significant results are reported at *p* < 0.05, and effect sizes for linear mixed effects models were calculated as outlined by Brysbaert and Stevens [91] and Westfall and colleagues [92].

#### MEG connectivity data

We used the Network-Based Statistic toolbox [93] to identify broadly distributed networks in theta, alpha, and beta that were specifically recruited for inhibitory control, relative to our control condition. We performed planned comparison *t* tests to detect networks that showed increased connectivity in the Inhibition vs. Vigilance condition in each group (Control, Inhibition > Vigilance; ASD, Inhibition > Vigilance), as well as between groups (ASD < Control, Inhibition > Vigilance; ASD > Control, Inhibition > Vigilance). To determine these networks, the *t* tests were applied at each pairwise connection and thresholded at values exceeding *t* = 2.708 (equivalent to *p* < 0.005). The largest network of contiguous suprathreshold connections was then subjected to permutation testing (5000 permutations), whereby an empirical null distribution of maximal network size was established by shuffling group labels. A family-wise error (FWE) corrected *p* value was calculated, signifying the probability of finding a network of equivalent or greater size, given the number of permutations, if the null hypothesis were true. Significant networks are reported at *p*_FWE_ < 0.05 and visualized using BrainNet Viewer [94].

#### Brain–behaviour relations

We explored the relations between any networks showing significant group differences and participants’ everyday inhibitory control, ASD symptom severity, and task performance. Specifically, we tested whether mean network connectivity values in these networks would predict any of these measures, and whether group status moderated this effect. Self-rated scores on the Inhibit scale of the BRIEF-A were used as an estimate of inhibitory control. SRS-2 self-rated Total scores were taken as an indication of severity of ASD symptoms. Task performance was determined using d′ scores. Analyses were performed in R 3.5.0 (R Core Team, https://www.r-project.org/). Significant results are reported at *p* < 0.05. Both unstandardized and standardized regression coefficients are reported as measures of effect size.

## Results

### BRIEF-A ratings

**Fig. 2:**
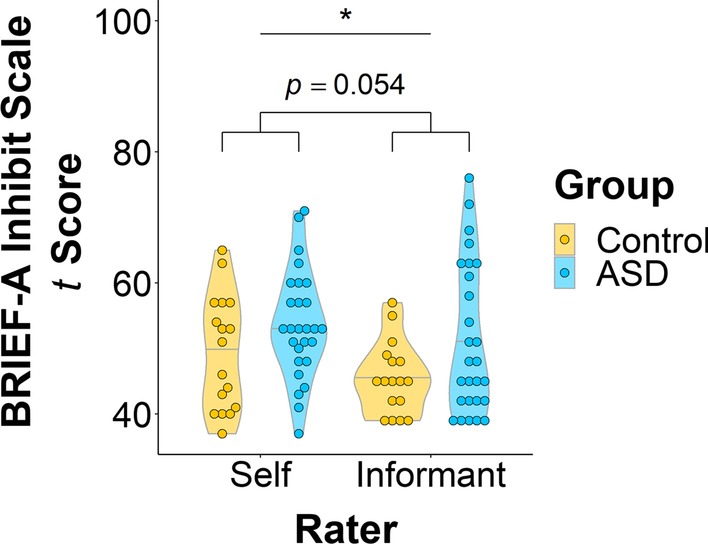
*T* scores on the Inhibit scale of the Behavior Rating Inventory of Executive Function, Adult Version (BRIEF-A). Only the difference in raters’ scores was statistically significantly different (*F*(1,40) = 7.17, *p* = 0.011, *d* = 0.13). There was a small, but nonsignificant difference between groups (*F*(1,39) = 3.95, *p* = 0.054, *d* = 0.25). * *p* < 0.05

While adults with ASD were rated by both themselves and their informants overall on the BRIEF-A as having more difficulties with inhibition compared to controls, this main effect of group across both self and informant ratings was only trending toward significance (*F*(1,39) = 3.95, *p* = 0.054, *d* = 0.25). However, scores significantly differed between raters (*F*(1,40) = 7.17, *p* = 0.011, *d* = 0.13), such that participants reported having more inhibitory control problems than their informants did (Fig. 2). Both self and informant ratings on the BRIEF-A are described in Table 2.

**Table 2:** Questionnaire self and informant ratings

	Control (*N *= 39)	ASD (*N *= 40)
	Mean (SD)	Mean (SD)
*BRIEF-A Inhibit Scale t Score*		
Self	49.47 (8.74) Range = [37–65], *n *= 17	53.63 (8.19) Range = [37–71], *n *= 27
Informant	45.47 (5.43) Range = [39–57], *n *= 17	51.30 (11.39) Range = [39–76], *n *= 27
*SRS-2 Total t Score*		
Self	48.63 (9.00) Range = [36–72], *n *= 16	66.71 (9.69) Range = [51–90], *n *= 28
Informant	43.94 (6.79) Range = [36–59], *n *= 16	64.82 (10.93) Range = [48–86], *n *= 28

*BRIEF-A *Behavior Rating Inventory of Executive Function, Adult Version, *SRS-2 *Social Responsiveness Scale, Second Version

### Task performance

**Fig. 3:**
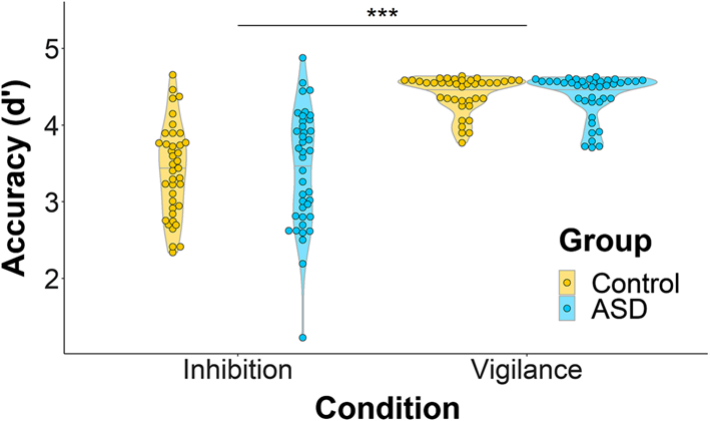
Accuracy on the Go/No-go task. There was a main effect of condition, where accuracy was poorer in the Inhibition than the Vigilance condition (*F*(1,74) = 197.81, *p* < 0.0001, *d* = 1.02). For control adults, mean accuracy was 82.72 ± 7.37% in the Inhibition condition and 99.44 ± 1.04% in the Vigilance condition. For adults with ASD, mean accuracy was 83.31 ± 8.32% in the Inhibition condition and 99.18 ± 1.26% in the Vigilance condition. *** *p* < 0.001

Adults with and without ASD performed equally well on the Go/No-go task (*F*(1,72) = 0.044, *p* = 0.84, *d* = 0.002), measured by d′. Both groups showed decreased accuracy during the Inhibition condition compared to the Vigilance condition (*F*(1,74) = 197.81, *p* < 0.0001, *d* = 1.02; Fig. 3).

### Neuroimaging

#### Within-group results

**Fig. 4:**
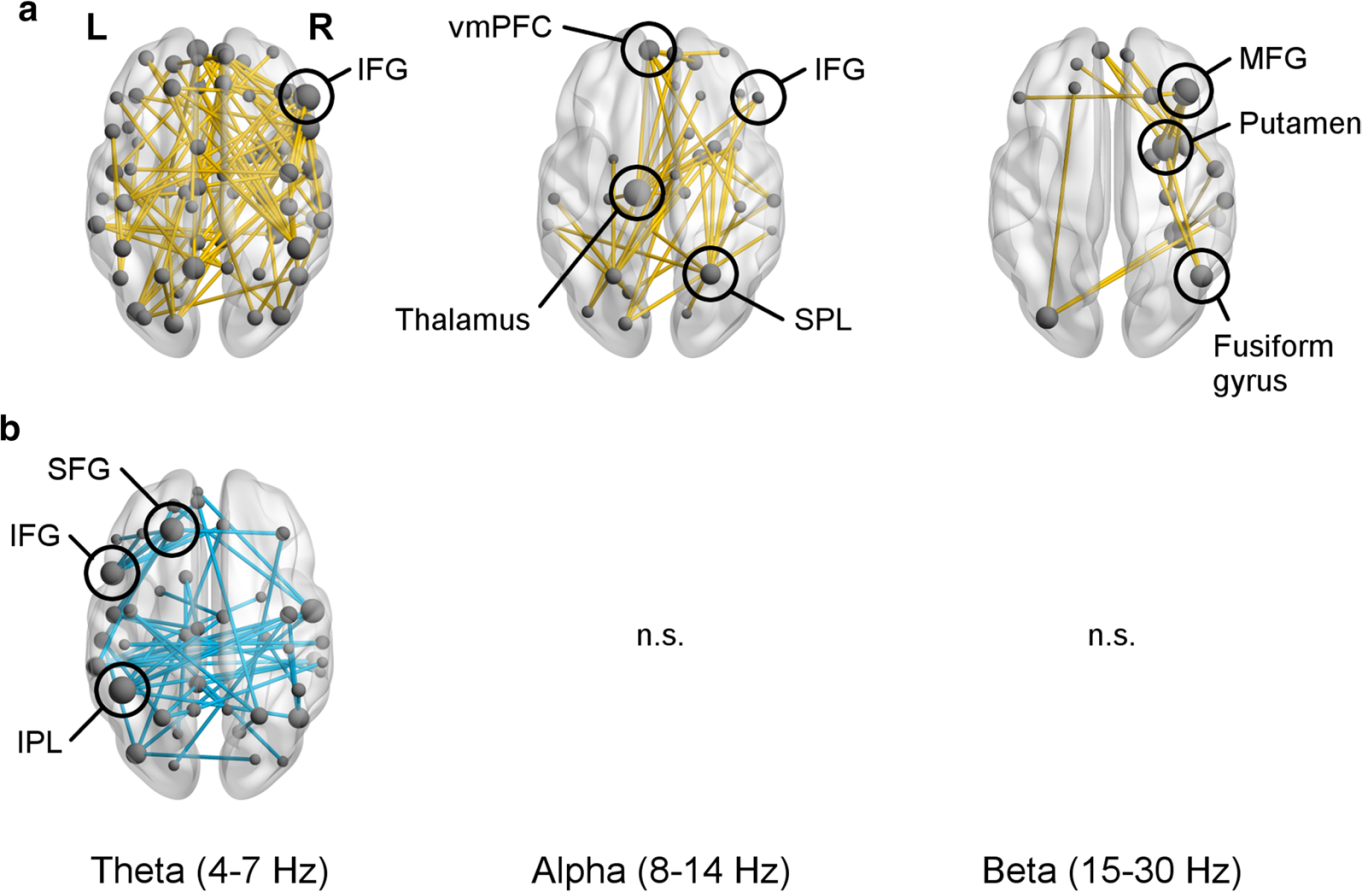
Networks of increased connectivity in **a** control adults and **b** adults with autism spectrum disorder (ASD) for correct No-go trials in the Inhibition compared to the Vigilance condition between 0 and 400 ms, post stimulus onset. **a** Control adults showed significantly increased network connectivity in the theta (*p*_FWE_ < 0.001), alpha (*p*_FWE_ = 0.004), and beta (*p*_FWE_ = 0.037) bands, while **b** adults with ASD only demonstrated greater network connectivity in the theta band (*p*_FWE_ < 0.001). Note that nodes are scaled by relative degree, or number of connections. The range of mean connectivity values in each network are detailed in Additional file 2: Table S1

Control adults displayed greater functional connectivity in the Inhibition than the Vigilance condition in the theta, alpha, and beta frequency bands between 0–400 ms (Fig. 4a). In the theta band, a broadly distributed network with a main hub (i.e., having a high number or degree of connections) in the right IFG was recruited (*p*_FWE_ < 0.001). A network in the alpha band with hubs in the left thalamus, left ventromedial prefrontal cortex (vmPFC), and right SPL, and which involved the right IFG, was also engaged (*p*_FWE_ = 0.004). A right-lateralized network in the beta band was additionally recruited (*p*_FWE_ = 0.037), with the right middle frontal gyrus (MFG), fusiform gyrus, and putamen showing the greatest degree.

Adults with ASD only showed greater connectivity for the Inhibition versus Vigilance condition in a network in the theta band (*p*_FWE_ < 0.001; Fig. 4b). Regions that had high degrees were the left IPL, left superior frontal gyrus (SFG), and left IFG. There were no significant findings in the ASD group in either the alpha (*p*_FWE_ = 0.617) or beta (*p*_FWE_ = 0.534) band.

#### Between-group results

**Fig. 5:**
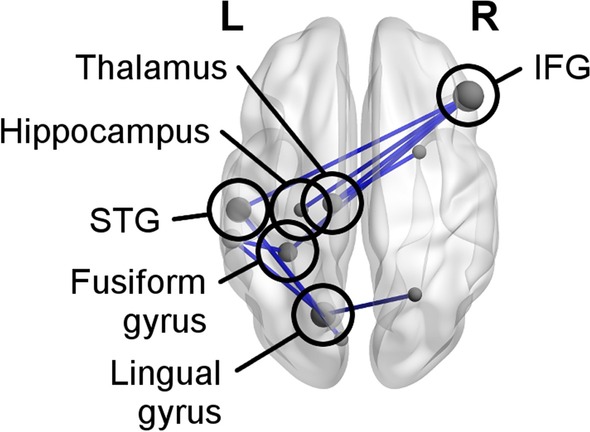
Network of regions showing connectivity differences between the ASD and control groups, occurring between 0 and 400 ms, post No-go stimulus onset in the Inhibition condition over the Vigilance condition. Adults with ASD had significantly (*p*_FWE_ = 0.038) decreased alpha-band connectivity compared to controls. Note that nodes are scaled by relative degree, or number of connections. The nodes in this network are reported in Additional file 3

Adults with ASD demonstrated decreased connectivity, compared to controls, in a network in the alpha band between 0–400 ms for the Inhibition condition relative to the Vigilance condition (Fig. 5; *p*_FWE_ = 0.038), such that mean connectivity in this network in the Inhibition condition was lower in adults with ASD than controls. The node with the highest degree in this network was the right IFG, which showed decreased connectivity with the left superior temporal gyrus (STG), fusiform gyrus, thalamus, and hippocampus. Adults with ASD did not show greater connectivity than controls in the alpha band, and the two groups did not differ significantly in terms of connectivity in either direction in the theta or beta band (all *p*s_FWE_ > 0.05).

### Brain–behaviour relations

**Fig. 6:**
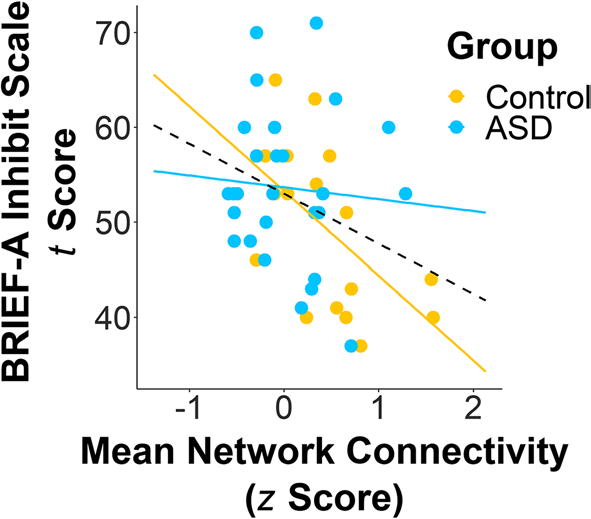
Relationship between mean network connectivity values (*z* scores) in the alpha band in the Inhibition condition of the Go/No-go task and Behavior Rating Inventory of Executive Function, Adult Version (BRIEF-A) self-reported *t* scores on the Inhibit scale. Mean alpha-band network connectivity inversely predicted BRIEF-A Inhibit scale scores (*b* = −5.09, *B* = −0.33, *p* = 0.042), such that participants who had greater connectivity in this network when inhibiting a prepotent response reported fewer problems with inhibition in everyday life. Mean network connectivity values were derived from the network in which adults with autism spectrum disorder (ASD) demonstrated decreased connectivity compared to controls between 0 and 400 ms, post stimulus onset, for the Inhibition greater than Vigilance condition in the alpha band. Solid lines represent the regression line for each group, while the dashed line signifies the regression line for the sample as a whole

Mean network connectivity values during the Inhibition condition in the alpha-band network showing significantly decreased connectivity in the ASD group negatively predicted self-rated scores on the Inhibit scale of the BRIEF-A (*b* = −5.09, *B* = −0.33, *p* = 0.042; Fig. 6). That is, greater connectivity in this network during response inhibition was associated with lower ratings on the Inhibit scale, or fewer self-reported issues with inhibitory control. There was no moderating effect of group on this relationship (*b* = −3.84, *B* = 0.25, *p* = 0.121). Neither mean connectivity in this network nor its interaction with group was significantly predictive of self-rated Total scores on the SRS-2 or task performance (all *p*s > 0.05; see Additional file 4: Fig. S1 for the association between mean network connectivity and SRS-2 self-rated Total scores).

## Discussion

The current study revealed that while adults with ASD exhibited no behavioural differences in an experimental Go/No-go task, there was a tendency for them to report experiencing difficulties with inhibition in everyday life on the BRIEF-A. Although behavioural performance on the Go/No-go task did not differentiate inhibitory control in each group, and the disparity in real-life inhibition between adults with and without ASD was small, patterns of brain connectivity related to inhibition in adults with ASD remained distinct from those without ASD.

At the group level, control adults activated networks in the theta, alpha, and beta bands, whose hubs mainly resided in the midline and right hemisphere, whereas adults with ASD only recruited a somewhat left-lateralized network in the theta band. Major nodes of the theta-band network in the ASD group comprised left homologues of brain regions implicated in inhibition, such as the IFG and IPL. Previous work has found that increases in theta power in left-lateralized regions of the inhibition network occur when resolving conflicting information during response inhibition [95]. While power and phase synchrony are not necessarily linked [96–98], the left-lateralization of this theta-band network in adults with ASD may reflect greater conflict between Go and No-go responses in the ASD group, leading to more effortful resolution of these opposing responses. Furthermore, given that individuals with ASD have demonstrated atypical lateralization of language [99, 100] and motor [101, 102] networks, it may not be surprising that they also show a more left-lateralized configuration of inhibitory control networks. Furthermore, a recent review of studies using MEG and EEG to examine functional brain connectivity in ASD [61] observed generally greater left lateralization of brain networks, which the authors interpreted as decreased integration of information between brain regions due to altered long-range connectivity typically found in the right hemisphere. Therefore, the left lateralization of the hubs of the theta-band network in our sample of adults with ASD may reflect altered communication among brain regions in the right hemisphere involved in inhibition, leading to atypical organization of homologous regions in the left hemisphere for inhibitory processes.

A similar narrative of reduced right hemisphere connectivity emerged when we compared the two groups directly: in the alpha band, adults with ASD showed decreased connectivity in a network with its main hub in the right IFG. In particular, the right IFG was less synchronized with other brain regions in the midline and left hemisphere, such as the thalamus, STG, and fusiform gyrus. This decrease in connectivity between the right IFG and left hemisphere areas may reflect diminished interhemispheric connectivity, as inter-areal alpha-band connectivity has been linked to top-down processing and coordination of distant brain regions [37, 50–52], and since the structure of the corpus callosum, which links the two hemispheres, has been often reported as impaired in individuals with ASD across development [103–107].

Whereas control adults demonstrated increased alpha-band connectivity between these areas for the Inhibition versus Vigilance conditions, adults with ASD showed no differences in alpha-band synchrony for this same comparison and very little activation of the alpha-band network that differed between groups during the Inhibition condition (Fig. 6). This finding implies that in the ASD group, the right IFG is less effective at modulating communication with other brain regions in the alpha band for successful response inhibition. Our results complement those of Kenet and colleagues [70], who also observed decreased alpha-band connectivity between brain regions involved in an antisaccade task, which they suggested implied that top-down mechanisms were impaired in adults with ASD. Although it is still unclear whether alpha-band synchrony is involved in recruiting task-relevant areas or suppressing task-irrelevant areas [50], as the right IFG has been shown consistently to be involved in response inhibition [19, 108–110], and as the other regions in this network have not been reliably associated with inhibitory control, it is likely that this deficit in alpha-band synchrony may indicate difficulty in constraining task-irrelevant activity. Moreover, given that we did not find any group differences in long-range synchrony of theta or beta oscillations, which have been associated with monitoring conflicting responses [32–34] and motor inhibition [38–40], respectively, our results suggest that these functions may be preserved in adults with ASD, and that their disparities in inhibitory control may be particular to the suppression of task-irrelevant information that is facilitated by alpha-band synchrony.

Considering that greater connectivity in this alpha-band network during the Inhibition condition was correlated with reports of better inhibitory control on the BRIEF-A, we propose that this decreased capability for inhibiting extraneous information impedes efficient top-down processing, ultimately leading to problems with inhibition in everyday life. Since participants completed our Go/No-go task in a very controlled and quiet environment, there were few distractors hindering task performance, but the degree of interference experienced in real-world situations from one’s surroundings is much greater, hence the dissociation between the behavioural results on our Go/No-go task and those on the BRIEF-A. This hypothesis is in line with research suggesting that individuals with ASD also experience difficulties with interference control [2, 4, 10], and that the right IFG is also involved in selecting appropriate strategies to achieve complex task goals [111, 112], such as those encountered in everyday life. Reduced top-down processing and inhibitory control may also contribute to the restricted, repetitive behaviours that are characteristic of ASD, as deficits in inhibition in ASD have been associated with greater severity of repetitive behaviours [113–116]. Thus, decreased alpha-band connectivity may influence not only interference control, but also the core symptom of repetitive behaviours in ASD.

## Limitations

As we were unable to obtain BRIEF-A and SRS-2 scores for all participants, our findings involving either of these measurements do not necessarily characterize our entire sample, especially in the context of our brain-behaviour analyses. With regard to the SRS-2 Total scores in particular, we may have not found any significant relations between it and mean connectivity, as there may have been other variables contributing to ASD symptom severity, such as deficits in social abilities and other executive functions. In addition, the difference between adults with and without ASD in scores on the Inhibit scale of the BRIEF-A was only significant at a trend level, which may have been partly due to the informants reporting fewer inhibition difficulties in our sample compared to participants’ own ratings. This small difference in inhibition skills indicates that our sample of adults with ASD had relatively preserved inhibitory control, especially as participants who performed poorly on the Go/No-go task had to be excluded from the analyses due to insufficient data. Adults with ASD in our study were also matched on IQ with the control group, so our findings may not necessarily extend to the larger ASD population that shows a wide range of cognitive abilities, especially those who experience more difficulties with inhibition. Finally, many of our reported effect sizes were in the small to medium range. Taken together, these limitations warrant future replication of our results in an independent, larger group of adults with ASD.

Although individuals with ASD show heterogeneity in functional connectivity patterns [117–120], our sample size was not large enough to examine this factor in the context of inhibitory control in ASD, which may explain why we did not observe recruitment of any alpha- or beta-band networks in the ASD group. Given that people with ASD may utilize alternative networks during inhibition, it would be important for future work to investigate whether adults with ASD demonstrate heterogeneity in the networks they recruit for inhibitory control. The theta, alpha, and beta frequency bands have all been implicated in different aspects of inhibition, and in our control group, all bands showed significant activity with this task. However, as this study is the first to look at whole-brain connectivity during response inhibition in adults with and without ASD and was therefore fairly exploratory, we did not perform corrections for the multiple tests conducted over the three frequency bands. Our main finding of decreased alpha-band connectivity in the ASD group would not have survived such a correction. However, the overlap in the specificity of our findings and those of others [70] to the alpha band suggests that adults with ASD demonstrate consistent differences in alpha-band connectivity related to inhibition.

This study also did not consider the effect of timing on functional connectivity. We averaged connectivity over 0–400 ms and compared the frequency-specific networks elicited during this time period between adults with and without ASD. However, there is evidence that connectivity is dynamic and therefore changes over time [121–124], and that individuals with ASD demonstrate differences in dynamic functional connectivity [125–128]. In addition, we performed an exploratory analysis of the event-related fields generated in response to No-go trials in the Inhibition condition, which revealed that adults with ASD may show some delay in this signal. We specifically observed that the peaks at 200 and 300 ms that have been consistently reported in the response inhibition literature and which have been shown to differ in ASD [129–133], were delayed in the ASD group by ~ 50 ms (Additional file 5: Fig. S2). Therefore, it would be critical for future work to characterize the evolution of the networks recruited for inhibitory control over time in adults with ASD.

## Conclusion

Overall, our study demonstrates that adults with ASD show atypical recruitment of brain networks during inhibitory control due to altered connectivity of right hemisphere regions typically involved in inhibition. We suggest that the lack of alpha-band connectivity observed in our ASD group, compared to our control group, implies reduced inhibition of task-irrelevant information by the right IFG. Since there was likely limited interference from extraneous stimuli in the Go/No-go task, the effects of this difficulty may be minimal during task performance. However, in complex, cognitively-demanding real-life situations, this decreased ability to suppress distractors may be more apparent and therefore interfere with their ability to exert inhibitory control, as seen in the association between lower alpha-band synchrony during prepotent response inhibition and poorer self-reported inhibition in everyday life. Future work should investigate whether adults with ASD also show atypical brain connectivity during tasks involving interference control, as impairments in this aspect of inhibitory control may account more precisely for the difficulties with inhibition that adults with ASD typically experience.

## Supplementary information

**Additional file 1:** A detailed description of how stimulus and ISI durations were adapted to participant performance.

**Additional file 2: Table S1.** Range of mean connectivity values (*z* scores) in networks recruited in the Inhibition > Vigilance condition in control and ASD groups.

**Additional file 3:** A list of brain regions in the network demonstrating significant differences between the control and ASD groups in the alpha band.

**Additional file 4: Figure S1.** A graph illustrating the relationship between mean network connectivity in the alpha-band network that was significantly different between groups and SRS-2 self-rated Total *t* scores.

**Additional file 5: Figure S2.** A plot of the event-related fields elicited by the control and ASD groups during correct No-go trials in the Inhibition condition.

## Data Availability

The datasets used and/or analysed during the current study are available from the corresponding author on reasonable request.

## Abbreviations

ACC: Anterior cingulate cortex
ADOS: Autism Diagnostic Observation Schedule
ASD: Autism spectrum disorder
BRIEF-A: Behavior Rating Inventory of Executive Function, Adult Version
EEG: Electroencephalography
FWE: Family-wise error
IFG: Inferior frontal gyrus
IPL: Inferior parietal lobule
ISI: Interstimulus interval
MEG: Magnetoencephalography
MFG: Middle frontal gyrus
SFG: Superior frontal gyrus
SMA: Supplementary motor area
SPL: Superior parietal lobule
SRS-2: Social Responsiveness Scale, Second Edition
STG: Superior temporal gyrus
vmPFC: Ventromedial prefrontal cortex
WASI: Wechsler Abbreviated Scale of Intelligence
wPLI: Weighted phase lag index
