# Supplementary material for: Alpha connectivity and inhibitory control in adults with autism spectrum disorder
Source: Mol Autism. 2020 Dec 7;11:95. doi: 10.1186/s13229-020-00400-y (PMC7722440; doi:10.1186/s13229-020-00400-y)
Supplement: Supplementary file 1 — Additional file 1: A detailed description of how stimulus and ISI durations were adapted to participant performance. [file 13229_2020_400_MOESM1_ESM.docx]

**Description of stimulus and ISI duration adaptation**

Stimulus duration would speed up by one frame (i.e., 16.67 ms on a computer with a 60 Hz refresh rate) if response time was less than the stimulus duration for at least four of the last five Go trials and the participant correctly did not respond on any of the last five No-go trials. It would also decrease by one extra frame if the participant had an overall No-go accuracy of ≥80%. Stimulus duration would slow down by one frame if response time was greater than the stimulus duration for three of the last five Go trials, or if the participant responded to two or more of the last five No-go trials and they had an overall No-go accuracy of <80%. ISI duration would speed up by one frame if participants refrained from responding on at least four of the last five No-go trials, overall No-go accuracy was ≥80%, and overall Go accuracy was ≥95%. It would slow down by two frames if overall Go accuracy was <95%, the participant responded to two or more of the last five No-go trials, or overall No-go accuracy was <80%. If both the latter two occurred, then the ISI would be increased by an additional four frames.
