# Supplementary material for: Alpha connectivity and inhibitory control in adults with autism spectrum disorder
Source: Mol Autism. 2020 Dec 7;11:95. doi: 10.1186/s13229-020-00400-y (PMC7722440; doi:10.1186/s13229-020-00400-y)
Supplement: Supplementary file 2 — Additional file 2: Table S1. Range of mean connectivity values (z scores) in networks recruited in the Inhibition > Vigilance condition in control and ASD groups. [file 13229_2020_400_MOESM2_ESM.docx]

**Table S1**

*Range of mean connectivity values (z scores) in networks recruited in the Inhibition > Vigilance condition in control and ASD groups*

|  | **Theta (4–7 Hz)** | **Alpha (8–14 Hz)** | **Beta (15–30 Hz)** |
| --- | --- | --- | --- |
| **Control (*N* = 39)** |  |  |  |
| Inhibition | 0.58–4.54 | -0.10–1.84 | -0.46–1.00 |
| Vigilance | -0.46–0.78 | -0.96–0.30 | -0.73–0.56 |
| **ASD (*N* = 40)** |  |  |  |
| Inhibition | 0.30–3.15 | --- | --- |
| Vigilance | -0.99–1.36 | --- | --- |
