# Supplementary material for: Alpha connectivity and inhibitory control in adults with autism spectrum disorder
Source: Mol Autism. 2020 Dec 7;11:95. doi: 10.1186/s13229-020-00400-y (PMC7722440; doi:10.1186/s13229-020-00400-y)
Supplement: Supplementary file 3 — Additional file 3: A list of brain regions in the network demonstrating significant differences between the control and ASD groups in the alpha band. [file 13229_2020_400_MOESM3_ESM.docx]

**Brain regions in the alpha-band network significantly different between control adults and adults with ASD**

The following regions were implicated in this network:

- Right Inferior Frontal Gyrus
- Left Hippocampus
- Left Calcarine Sulcus
- Left Lingual Gyrus
- Left Fusiform Gyrus
- Right Superior Parietal Lobule
- Right Putamen
- Left Thalamus
- Left Superior Temporal Gyrus
- Left Middle Temporal Gyrus
