# Supplementary material for: Alpha connectivity and inhibitory control in adults with autism spectrum disorder
Source: Mol Autism. 2020 Dec 7;11:95. doi: 10.1186/s13229-020-00400-y (PMC7722440; doi:10.1186/s13229-020-00400-y)
Supplement: Supplementary file 4 — Additional file 4: Figure S1. A graph illustrating the relationship between mean network connectivity in the alpha-band network that was significantly different between groups and SRS-2 self-rated Total t scores. [file 13229_2020_400_MOESM4_ESM.docx]

**Figure S1**


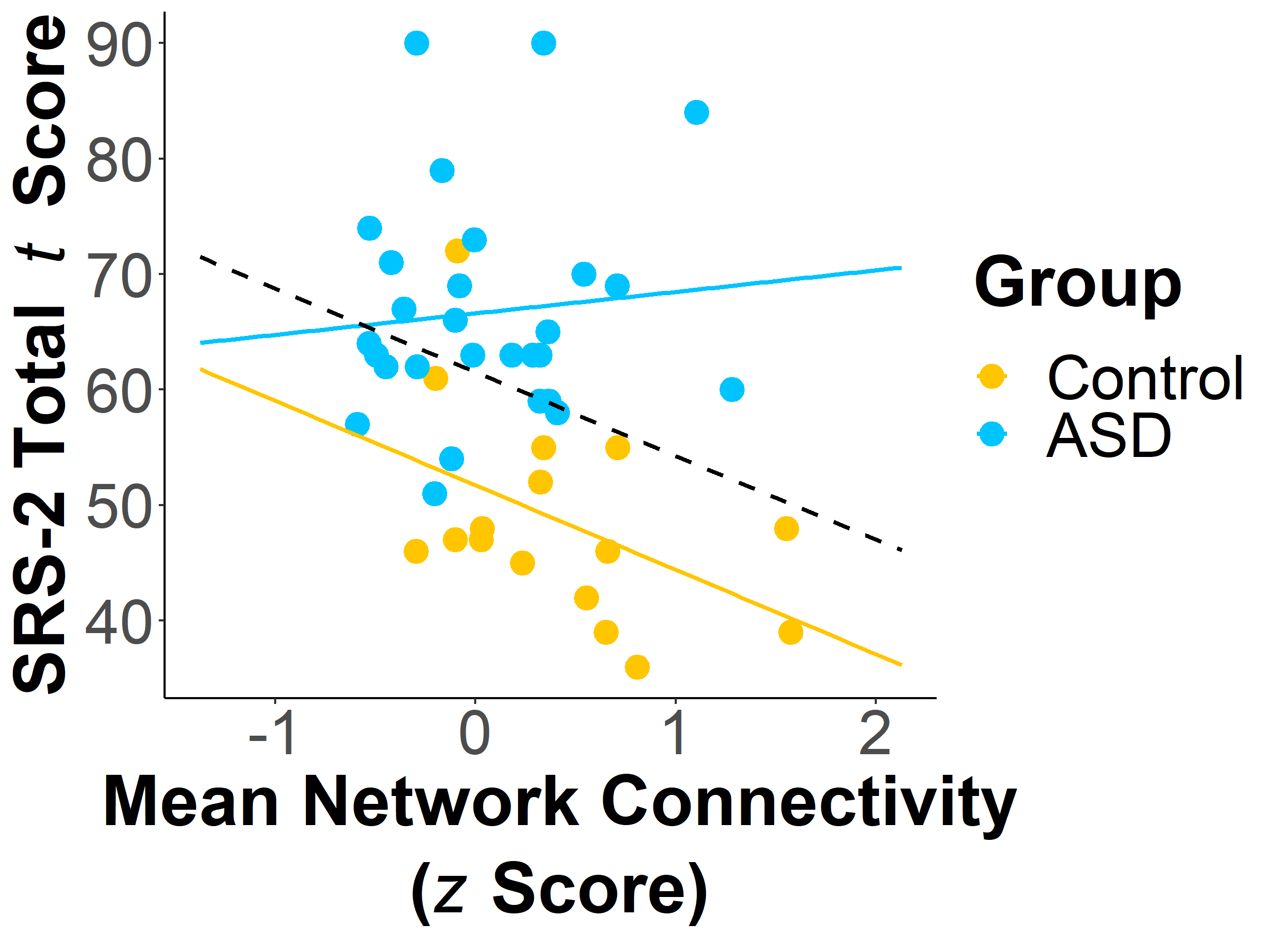


**Fig. S1** – Relationship between mean network connectivity values (*z* scores) in the alpha band in the Inhibition condition of the Go/No-go task and Social Responsiveness Scale, Second Version (SRS-2) self-rated Total *t* scores. There was no significant effect of mean network connectivity nor an interaction with group on SRS-2 self-rated Total scores (all *p*s > 0.05).
