# Supplementary material for: Alpha connectivity and inhibitory control in adults with autism spectrum disorder
Source: Mol Autism. 2020 Dec 7;11:95. doi: 10.1186/s13229-020-00400-y (PMC7722440; doi:10.1186/s13229-020-00400-y)
Supplement: Supplementary file 5 — Additional file 5: Figure S2. A plot of the event-related fields elicited by the control and ASD groups during correct No-go trials in the Inhibition condition. [file 13229_2020_400_MOESM5_ESM.docx]

**Figure S2**


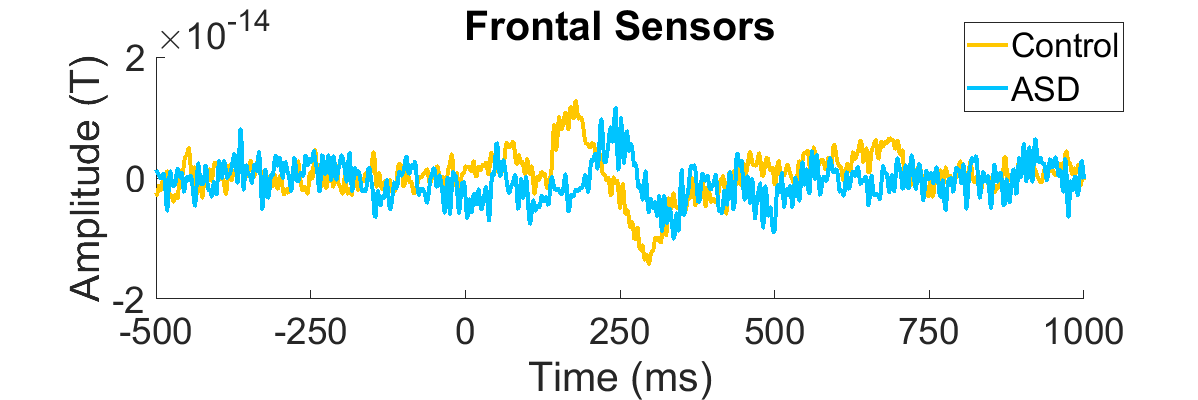


**Fig. S2** – Event-related fields elicited during the Inhibition condition. After preprocessing, the MEG signal from all frontal sensors was averaged over all correct No-go trials in the Inhibition condition. They were then grandaveraged over all participants and baselined from -500 to 0 ms to generate the event-related fields plotted here. The control group demonstrated peaks in the event-related fields at around 200 and 300 ms, while the ASD group showed peaks at approximately 250 and 350 ms.
